# Editorial: How, and Why, Does Spatial-Hearing Ability Differ among Listeners? What is the Role of Learning and Multisensory Interactions?

**DOI:** 10.3389/fnins.2016.00036

**Published:** 2016-02-16

**Authors:** Guillaume Andéol, Brian D. Simpson

**Affiliations:** ^1^Département Action et Cognition en Situation Opérationnelle, Institut de Recherche Biomédicale des ArméesBrétigny sur Orge, France; ^2^Air Force Research LaboratoryDayton, OH, USA

**Keywords:** sound localization, individual differences, cocktail party, learning, training, HRTF, multisensory perception, spatial hearing

Large individual differences are relatively common in human perception. Spatial hearing is not an exception; for instance, two listeners can perceive the same auditory target to be at very different spatial locations. Such variability cannot be considered as mere experimental noise but as true data that we have to use for explaining the mechanisms underlying the perception of auditory space. The 22 papers of this research topic explore individual differences in almost every aspect of auditory space perception.

To determine the position of a sound source on a Left/Right axis (from −90° at the extreme left to +90° at the extreme right), listeners use binaural cues: interaural differences in level (ILDs) and in time (ITDs). The auditory system is sensitive to ITDs for stimuli below about 1500 Hz, but this sensitivity declines rapidly at higher frequencies. It has been suggested that this reduced sensitivity at higher frequencies acts as a protective mechanism against ambiguous information that results from the similarity between head radius and the wavelengths for these frequencies. By providing quantitative data, Hartmann and Macaulay reconsidered this explanation and showed that this mechanism would only be effective for heads that are 50% smaller than current adult heads. The authors presented potential developmental and evolutionary processes that could explain how ILDs would replace ITDs for the localization of frequencies above 1500 Hz. Ochi et al. found that individual differences in ITDs and ILDs sensitivities were related to basic non-spatial abilities (i.e., the efficiency of temporal coding for the ITDs and of intensity coding for the ILDs). Gallun et al. reported that temporal coding was influenced both by hearing loss and aging, but these factors were independent. The authors reached this conclusion by applying a linear mixed model on a population of 78 listeners with a large range of hearing thresholds and ages. In older adults, Perez et al. showed that temporal coding could partially predict satisfaction of hearing impaired listeners recently fitted with hearing aids. Specifically, those with better abilities prior to fitting were less satisfied after fitting, presumably due to higher, and so unfulfilled, expectations. In a special case of hearing impairment, single-sided deafness, listeners retain some localization abilities in azimuth, even in the absence of normal binaural cues, but large individual differences are observed (Agterberg et al.). It seems that listeners can use the direction-dependent modifications in the spectrum of the incoming sound wave induced mainly by the outer ears for localization in azimuth, whereas normal hearing listeners preferentially use them for localization in the up/down and front/back dimensions. These so-called spectral cues are restricted to the high-frequency region (above approximately 4 kHz) because of the limited physical dimensions of the outer ears. Interestingly, the authors found that an individual's localization performance was related to high-frequency thresholds at that individual's hearing ear.

All the acoustical transformations of the incoming soundwave that occur before reaching the tympanum can be captured by the head-related transfer function (HRTF; see Wightman and Kistler, 1989). Due to obvious anatomical differences, HRTFs vary substantially across listeners. By decomposing the HRTF into several non-directional and directional components, Romigh and Simpson demonstrated that the perceptually relevant differences between sets of HRTFs are mainly restricted to the components containing the spectral cues. According to Alves-Pinto et al., the recovery of spectral cues depends on temporal coding, mainly operated by low- and medium-spontaneous-rate fibers of the auditory nerve. Therefore, the individual differences often observed in localization judgments in the Up/Down and Front/Back dimensions could be explained by the state of functioning of these fibers, which may be altered in noise-exposed listeners, even if they have normal audiometric thresholds. In order to mitigate the risk of noise-induced hearing loss, listeners can choose among a large range of hearing protection devices. Zimpfer and Sarafian showed that such devices disturbed localization, particularly in the Up/Down and Front/Back dimensions, but differently depending on the device. Measurements of the alterations of the HRTFs of a manikin by the different devices could explain the observed variability in localization performance found across devices. Many studies have explored the mechanisms of adaptation after the alterations of localization cues induced by ear molds, hearing aids, or other means (e.g., Hofman et al., 1998). They showed that, after an initial degradation, and despite individual differences, localization performance improved for most listeners over time, approaching performance obtained with unaltered (natural) cues. These works were analyzed by two review articles in this research topic. Mendonça compared the methodological aspects of the studies, particularly the types and durations of training and their effects on adaptation. Carlile underlined the individual differences in adaptation and suggested that these differences could be attributed to (1) interactions with the environment during adaption, and (2) the degree to which the spectral cues were initially altered. He also pointed out the role played by auditory-motor learning in the adaptation process. Whereas, both Medonça and Carlile noted that multisensory training is more efficient than training auditorily only, Noel and Thelen indicated that cross-modal training (for instance, interleaved visual, and auditory training) could also facilitate adaptation to new spatial cues. They also remarked that cross-modal and multisensory training regimens could have different long-term effects that need to be clarified before their use for restorative care.

Improving sound localization performance can also occur with non-altered, normal spectral cues as showed by Andéol et al. Using perceptual training with visual feedback, listeners improved their performance proportionally to their pretraining score, which leads to a reduction of the individual differences. In this study involving naive listeners, the effects of using non-individual acoustic cues was moderate. Interestingly, Majdak et al. demonstrated that non-acoustic factors (such as perceptual abilities) were better predictors of sound localization performance than acoustic factors (such as the quality of the directional cues in the HRTFs), suggesting that the origins of the individual differences would be more perceptual than physical, at least for the judgment of source direction.

Beyond direction, localization implies the determination of source distance. Three articles tackled auditory distance perception in this research topic. Anderson and Zahorik examined distance perception in three conditions: auditory, visual, and auditory-visual. They found that distance judgments were most accurate, and less variable, across subjects in the visual and the auditory-visual conditions relative to the auditory-alone condition. The authors used a large range of target distances (from 0.3 to 9.7 m) but only one direction (straight ahead). The study of Parseihian et al. was therefore complementary to the study of Anderson and Zahorik in that they examined distance perception employing several target azimuths, but only in the near field (<1.08 m); nevertheless, they also observed significant individual differences and poor performance for auditory distance judgment. However, this performance can improve, as shown by Wisniewski et al. They found large effects of training on distance perception and, interestingly, they explained individual differences in the observed improvements by training-induced modifications in the activity of non-auditory cortical areas.

Most of the previously mentioned studies were performed in static conditions. Two studies included in this issue examined auditory space perception in a more natural condition (i.e., with a listener's head and/or the auditory stimulus in motion). Brimijoin and Akeroyd assessed a listener's ability to segregate two sources while the sources and/or the listener were moving. They found better performance with self-motion than with source motion. Interestingly, the individual differences they observed were not explained in terms of age or hearing loss. McAnally and Martin investigated the effect of head movements on source direction accuracy. They found better elevation and front/back judgments as the amplitude of head movements increased, with few individual differences.

In a multitalker speech recognition task, the spatial separation of talkers, as well as sex differences across talkers, could facilitate the understanding of speech (for a recent review see Bronkhorst, 2015). Zekveld et al. compared their relative effects on cognitive load using pupillary response and found that sex differences were more effective.

To act in a multisensory environment, an efficient multisensory representation of the external space needs to be achieved by multisensory integration. Godfroy-Cooper et al. assessed the precision and acuity of auditory, visual, and auditory-visual spatially-congruent targets in the frontal field. They showed that the target position influenced the relative perceptual weights assigned to each modality, even if vision dominated in most cases. Without vision, the representation of space could still be accurate, as demonstrated by Viaud-Delmon and Warusfel with an auditory version of the “Moris water maze” in blindfolded listeners. Blindfolding is often used to isolate the auditory spatial processes, but it should be done with caution. Indeed, Tabry et al. noticed that blinfolded listeners demonstrated biaises in their localization judgment for head pointing (but not for hand pointing).

Wightman and Kistler (1999) stated that individual differences in sound localization are “a source of both frustration and inspiration.” These differences have indeed inspired the articles included in this special issue, which provide exciting and up-to-date results in this area of growing interest. The studies included here demonstrate that many factors—physical, perceptual, and cognitive—play a role in individual differences in spatial hearing. Examining the interaction of these factors will help to provide insights that inform our understanding of the mechanisms underlying spatial hearing and how such mechanisms could produce such a diversity of behaviors.

## Author contributions

All authors listed, have made substantial, direct and intellectual contribution to the work, and approved it for publication.

### Conflict of interest statement

The authors declare that the research was conducted in the absence of any commercial or financial relationships that could be construed as a potential conflict of interest.
